# A comparison of routine [^68^Ga]Ga-PSMA-11 preparation using Locametz and Illuccix kits

**DOI:** 10.1186/s41181-024-00317-4

**Published:** 2024-12-18

**Authors:** Ivan E. Wang, Luke J. Morrissette, Ka Kit Wong, Allen F. Brooks, Marianna Dakanali, Peter J. H. Scott

**Affiliations:** 1 Department of Medicinal Chemistry, College of Pharmacy, University of Michigan, Ann Arbor, MI 48109 USA; 2 Department of Radiology, University of Michigan, 2276 Medical Science 1, 1301 Catherine St., Ann Arbor, MI 48109-5610 USA; 3 Department of Pharmacology, University of Michigan, Ann Arbor, MI 48109 USA

**Keywords:** Gallium-68, Gozetotide, Illuccix, Locametz, PET manufacturing, Prostate cancer, PSMA-11, Radiopharmacy

## Abstract

**Background:**

Approval of Locametz and Illuccix kits for the manufacture of [^68^Ga]Ga-PSMA-11 (gallium Ga68 gozetotide), a PET imaging agent for prostate cancer, as well as the corresponding therapeutic ([^177^Lu]Lu-PSMA-617 Pluvicto), has led to a rapid increase in demand for [^68^Ga]Ga-PSMA-11 PET imaging. Radiopharmaceutical manufacturers, using ^68^Ge/^68^Ga generators, may decide to adopt Locametz and/or Illuccix kits, which requires a comparison to select the most suitable kit for day-to-day use. The objective of this article is to compare both kits and provide guidance for selecting one for routine use, as well as evaluate labeling consistency of both kits during routine production. Additionally, we report our experience during 1.5 years of daily [^68^Ga]Ga-PSMA-11 production at our facility using both kits.

**Results:**

Locametz (n = 181) and Illuccix (n = 256) kits were prepared using non-silicone coated and silicone-coated needles with ^68^Ga activities ranging from 0.53 to 3.16 GBq, with a failure rate of 1 in 128 runs for both kits. With Locametz, a 3.7 GBq generator and 10-min incubation at room temperature gave doses that passed quality control (QC) testing. Use of non-silicone coated needles in the process led to solution discoloration, and QC failure. Additionally, lack of vial inversion led to inconsistent labeling, which improved with subsequent vial agitation. For Illuccix, addition of the acetate buffer to the precursor vial prior to adding the [^68^Ga]GaCl_3_ simplifies the workflow. The maximum tolerated activity was 1.85 GBq. Lack of vial inversion led to failures, which were rectified by agitating the vial to properly incorporate the acetate solution with the generator eluate.

**Conclusions:**

Both kits benefited from using a syringe pump to elute the ^68^Ge/^68^Ga generator, vial agitation, and longer length/smaller bore silicone coated needles. Both kits have similar workflows, comparable QC outcomes, and result in equivalent clinical images. Thus, the decision between kits will ultimately be determined by production preferences. Since radiopharmacies have an established “kit-based” workflow, Locametz kits with higher allowed activities and longer shelf-life may offer benefits. Conversely, more traditional PET manufacturing facilities might benefit from using Illuccix kits due to compatibility with cyclotron-produced [^68^Ga]GaCl_3_ allowing for kit batching. Ultimately, the commercial availability of 2 approved kits for production of [^68^Ga]Ga-PSMA-11 PET has facilitated ready access to this important new imaging agent.

**Supplementary Information:**

The online version contains supplementary material available at 10.1186/s41181-024-00317-4.

## Background

According to the American Cancer Society and the NIH Surveillance, Epidemiology, and End Results (SEER) data set, one in every eight men will be diagnosed with prostate cancer in their lifetime, with the average age of first diagnosis at 66 years old (Siegel et al. 2023). In the United States, prostate cancer is the second most common cancer type next to skin cancer, with estimates of about 288,000 new cases annually (Noone et al. 2018; National Cancer Institute 2024). High prevalence and diagnosis reliant on screening tests and symptoms, excluding a prostate biopsy which is the only true diagnosis but highly invasive, has led to the development of numerous imaging modalities to help diagnose and manage prostate cancer. These include transrectal ultrasound, magnetic resonance imaging, computerized tomography (CT) scans, and positron emission tomography (PET) (Zelefsky et al. 2019; Niederhuber et al. 2020; Taplin and Smith 2023). In patients with high disease burden and associated metastases, use of PET/CT scans is beneficial in identifying the location of metastasized tumors. Use of the prostate-specific membrane antigen (PSMA) as a target in prostate cancer imaging is well established and the ligand [^68^Ga]Ga-PSMA-11 (gallium Ga68 gozetotide), first reported by investigators at the German Cancer Research Center and Heidelberg University (Schäfer et al. 2012; Eder et al. 2013; Sachpekidis et al. 2016) is established for the PET imaging of prostate cancer today (Barrio et al. 2016; Clore and Scott 2024).

Labeling bioactive molecules with gallium-68 is unique, as unlike other common PET isotopes such as carbon-11 or fluorine-18, there are two major production methods: cyclotron irradiation of a ^68^Zn target (either liquid or solid), or elution of a germanium-68/gallium-68 (^68^Ge/^68^Ga) generator, to source the [^68^Ga]GaCl_3_ used in subsequent labeling steps. Both methods have their benefits and drawbacks. For instance, cyclotron-based production yields consistent supply and higher activity, but requires a more complicated purification process. In contrast, generator-based production allows straightforward access to ^68^Ga-labeled tracers in facilities without a cyclotron, but requires careful management of generator decay. Both methods have been used to prepare [^68^Ga]Ga-PSMA-11. For example, we and others produced [^68^Ga]Ga-PSMA-11 for use in early clinical trials under an FDA-approved investigational new drug (IND) application via both a generator approach using a Scintomics synthesis module, and utilizing cyclotron-produced ^68^Ga in conjunction with the GE FASTLab developer platform (Lin et al. 2018; Pandey et al. 2019; Rodnick et al. 2020, 2022; Jackson et al. 2020; Thisgaard et al. 2021; Svedjehed et al. 2022). Following successful completion of phase 3 clinical trials, the University of California Los Angeles (UCLA) and the University of California San Francisco (UCSF) submitted concomitant New Drug Applications for [^68^Ga]Ga-PSMA-11 to the FDA for marketing authorization. This was granted in December 2020, making [^68^Ga]Ga-PSMA-11 the first radiopharmaceutical for PET imaging of PSMA–positive lesions in men with prostate cancer approved in the US (Carlucci et al. 2021).

The pioneering efforts by UCSF and UCLA paved the way for commercial manufacturers to develop kits for the production of [^68^Ga]Ga-PSMA-11. Cold kit labeling is expected to facilitate global access to ^68^Ga-labeled radiopharmaceuticals including [^68^Ga]Ga-PSMA-11 (Lepareur 2022; van Brandwijk et al. 2024), and FDA approval of commercial kits for producing [^68^Ga]Ga-PSMA-11 has altered the way many sites produce [^68^Ga]Ga-PSMA-11 for routine clinical use. Illuccix, manufactured by Telix Pharmaceuticals, was approved in 2021, and Locametz, manufactured by Novartis, in 2022 (Novartis Pharmaceuticals Corporation 2022; Telix Pharmaceuticals 2023). These kits have also been approved by other regulatory agencies around the world. For traditional PET manufacturing facilities, approval of these kits has necessitated introduction of generator-based production into historically cyclotron-based workflows. In radiopharmacies and/or nuclear medicine departments already familiar with a kit-based workflow, approval of these kits has nevertheless increased the daily workload, and necessitated handling both PET (^68^Ga) and SPECT (e.g. ^99m^Tc) radionuclides with different storage requirements and elution timing due to differences in generator age and kinetics. In either instance, manufacturers need to decide on adoption of Illuccix and/or Locametz kits in the formulary, depending on which is more appropriate for routine use at a given site.

Approval of the Locametz and Illuccix kits with use of ^68^Ge/^68^Ga generators has enabled on demand access to [^68^Ga]Ga-PSMA-11 in radiopharmacies and nuclear medicine departments. Since both Locametz and Illuccix kits contain the same precursor, PSMA-11, but with differences in formulation or excipients and labeling procedure, there are questions to consider when selecting one kit over the other (see: Supplementary Information, Table S1). The focus of this article is to compare both kits, provide guidance for radiopharmaceutical manufacturers to help select the most fitting kit for daily production of PSMA-11 at their own facility, and to provide guidance for switching from one kit to another. Additionally, we highlight some difficulties that were experienced during a year and a half of routine [^68^Ga]Ga-PSMA-11 production at our facility, and solutions that we developed to overcome such issues.

## Materials and methods

### Introduction

Using E&Z GalliaPharm® ^68^Ge/^68^Ga generators (Eckert and Zigler 2021), Locametz (n = 181) and Illuccix (n = 256) kits were prepared at the University of Michigan from 2nd June 2022 to 13th September 2023. The workflow, variations in production batches, failure rates, and QC parameters were analyzed. QC tests were completed in accordance with kit package inserts, and according to standard procedures described in the [^68^Ga]Ga-PSMA-11 monograph in the European Pharmacopeia and general chapters of the United States Pharmacopeia (USP) (European Pharmacopeia 2021; United States Pharmacopeia 2024a,b).

Certain tests listed in the European Pharmacopeia monograph were omitted: ethanol and HEPES content was not determined as preparation of either Locametz or Illuccix does not involve ethanol or HEPES buffer in the process, and radioTLC according to the package inserts was used to determine purity rather than radioHPLC for operational simplicity. RadioHPLC can also give information on the ratio of [^68^Ga]Ga-PSMA-11 diastereoisomers but, since this is a known issue (US Food and Drug Administration 2022) and the diastereoisomers have similar affinities for PSMA (Eder et al. 2014), HPLC analysis is not required by the package inserts.

### Production of [^68^Ga]Ga-PSMA-11 using Locametz kits

#### Overview

The procedure for producing [^68^Ga]Ga-PSMA-11 using a Locametz kit in conjunction with an Eckert and Ziegler (E&Z) GalliaPharm® ^68^Ge/^68^Ga Generator, as used at our facility, is described in the package insert (Novartis Pharmaceuticals Corporation 2022). Briefly, [^68^Ga]GaCl_3_ is eluted from the generator using 5 mL of 0.1 M HCl directly into the Locametz vial fitted with a 0.2 μm sterile vent filter. The vial is then inverted once, placed upright, and left at room temperature (20–30 °C) for 5 min for labeling. The vial is then calibrated with a maximum allowable activity of 2.59 GBq and an expiration time of 6 h is assigned. The dose in 5 mL can be further diluted to 10 mL with normal saline (0.9% NaCl).

#### Detailed procedure

**Fig. 1:**
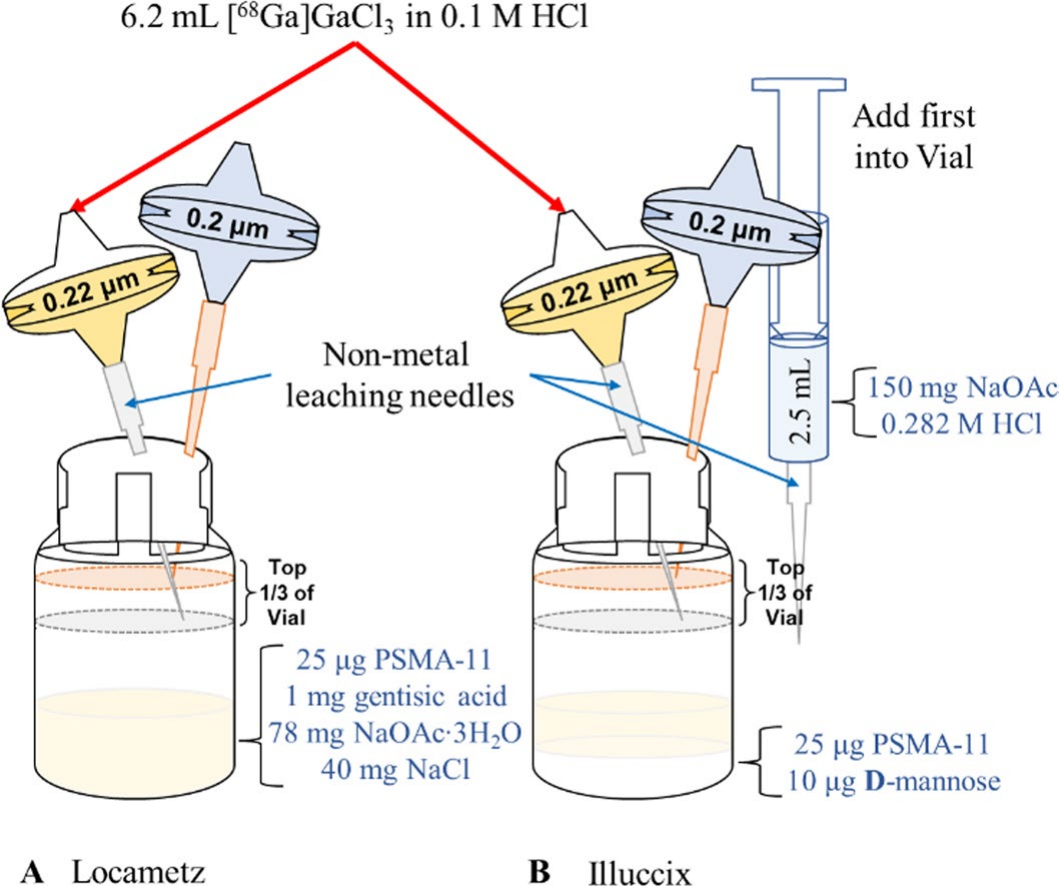
Visual depiction of labeling process for Locametz and Illuccix kits. The assembly and components of Locametz (**A**) and Illuccix, *configuration A* (**B**) vials prior to the addition of generator produced [^68^Ga]GaCl_3_ from an E&Z generator. Vial assembly occurs in a segregated area with an ISO 5 primary engineering control (PEC) with subsequent elution of a ^68^Ge/^68^Ga generator in an ISO 8 secondary engineering control (SEC). Non-metal leaching needles (NMLN) should be used to transfer the generator eluate into the dose vial. Abbreviations: NaOAc – anhydrous sodium acetate, NaOAc∙3H_2_O – Sodium Acetate Trihydrate

At our facility, production of Locametz kits requires the disposable and non-disposable items listed in Table S2 (see Supplementary Information). Production of Locametz occurs as follows: Take the Locametz vial from the kit and remove the flip-off cap from the top of the vial. Take a tear weight of the vial. In a segregated area with an ISO 5 primary engineering control (PEC), move and wipe the needed materials into the PEC using sterile 70% isopropyl alcohol. Using aseptic technique, pierce the Locametz dose vial with a 0.2 μm Millex-FG sterile air vent filter connected to a 0.8 × 50 mm (21Gx2″) needle. Then pierce the Locametz dose vial with a 0.22 μm Cathivex-GV sterile product filter connected to a 1.2 × 38 mm (18Gx1½”) silicone coated needle. Arrange the filters such that the bevel of the needle connected to the sterile air vent is positioned above the bevel of the needle connected to the product filter, and that both filters are in the top 1/3 of the Locametz vial (Fig. 1a). This will minimize activity from being expelled out of the sterile vent filter during generator elution. Take the Locametz dose vial with the two filters out of the PEC and place it into a lead or tungsten shielded container suitable for PET isotopes. Connect the 1.85 GBq or 3.7 GBq E&Z generator outlet line to the Cathivex-GV filter and using a 10 mL organic syringe pull up 6.2 mL of 0.1 M Ultrapure sterile HCl from the three-way valve upstream of the inlet line minimizing air bubbles. Manually (or automatically if using a syringe pump), elute the generator at a rate no faster than 2 mL/min, ensuring that if there were air bubbles in the syringe they are not pushed into the generator. Once the elution is completed, note the time, as this is used to determine the expiration time. Remove the sterile air vent from the Locametz vial and discard in a shielded radioactive sharps bin. Remove the product filter from the Locametz vial and disconnect the filter from the generator outlet line and place it into a lead or tungsten shielded container. This will be used for the filter integrity test. Using tongs and behind appropriate shielding (e.g. lead L-block), invert the Locametz vial twice and agitate vigorously (shake up, down, and left, right) for 2 s then place the vial back into the vial shield. Alternatively, the entire vial shield (and vial) can be inverted and agitated. Let the vial sit at room temperature (20–25 °C) for 10 min for labeling to take place (Fig. 1a).

#### QC testing

For QC testing, the preferred method for determining radiochemical purity (RCP) is using an instant thin layer chromatography (TLC) method with a radiometric TLC (rTLC) plate scanner (see below).

### Production of [^68^Ga]Ga-PSMA-11 using Illuccix kits

#### Overview

Production using Illuccix kits with E&Z generators (configuration A) is described in the corresponding Illuccix package insert (Telix Pharmaceuticals 2023). Briefly, [^68^Ga]GaCl_3_ is eluted from the generator using 5 mL of 0.1 M HCl into a sterile vacuumed reaction vial (vial 3) fitted with a 0.2 μm sterile vent filter to equalize pressure. With a 10 mL syringe connected to a needle, the acetate buffer (vial 2) is injected into the vial containing PSMA-11 (vial 1). Once combined, the mixture is swirled to dissolve the peptide in the buffer. Then, using a new 10 mL syringe, the buffer and ligand solution is added into the vial containing [^68^Ga]GaCl_3_ (vial 3). The resulting solution is left at room temperature (15–30 °C) for 5 min for labeling to occur. The vial is then calibrated, with a maximum allowable activity of 1.85 GBq, and an expiration time of 4 h is assigned. The dose will have a final volume of 7.5 mL.

#### Detailed procedure

At our facility, production of Illuccix kits requires the disposable and non-disposable items listed in Table S3 (see Supplementary Information). Production of Illuccix occurs as follow: Take vial 1 from the Illuccix kit and remove the blue flip-off cap from the top of the vial. Take a tear weight of the vial. In a segregated area with an ISO 5 PEC, move and wipe the needed materials into the PEC using sterile 70% isopropyl alcohol. Using aseptic technique, pierce vial 2 (acetate buffer vial) using a 5 mL organic syringe connected to a 1.2 × 38 mm (18Gx1½”) silicone coated needle and withdraw the entire contents of the vial (2.5 mL) minimizing the headspace in the syringe. Insert the syringe with the acetate buffer into vial 1 and inject the buffer solution into the vial. Vial 1 is under negative pressure (vacuum) and therefore does not need its pressure equalized. Remove the syringe and needle from vial 1 and agitate gently (side to side, not up and down) for 5 s. Then pierce vial 1 first with a 0.2 μm Millex-FG sterile air vent filter connected to a 0.8 × 50 mm (21Gx2″) needle, and second with a 0.22 μm Cathivex-GV sterile product filter connected to a 1.2 × 38 mm (18Gx1½”) silicone coated needle. Arrange the filters such that the bevel of needle connected to the sterile air vent is positioned above the bevel of the needle connected to the product filter and that both filters are in the top 1/3 of vial 1 (Fig. 1b). This will minimize activity from being expelled out of the sterile vent filter during generator elution. Take vial 1 with the two filters out of the PEC and place into a lead or tungsten shield suitable for PET isotopes. Connect the 1.85 GBq E&Z generator (or a 3.7 GBq E&Z generator decayed to only producing at maximum of 1.85 GBq) outlet line to the Cathivex-GV filter and using a 10 mL organic syringe pull up 6.2 mL of 0.1 M Ultrapure sterile HCl from the three-way valve upstream of the inlet line minimizing air bubbles. Manually (or automatically when using a syringe pump), elute the generator at a rate no faster than 2 mL/min, ensuring that if there were air bubbles in the syringe it is not pushed into the generator. Once the elution is completed, note the time, as this is used to determine the expiration time. Remove the sterile air vent from vial 1 and discard in a shielded radioactive sharps bin. Remove the product filter from vial 1 and disconnect the filter from the generator outlet line and place it into a lead or tungsten shield. This will be used for the filter integrity test. Using tongs and behind appropriate shielding (e.g. lead L-block), agitate the vial (shake up, down, and left, right) for 2 s then place the vial back into the vial shielded container. Alternatively, the entire vial shield (and vial) can be agitated. Let the vial sit at room temperature (15–30 °C) for 5 min for labeling to take place (Fig. 1b).

#### QC testing

For QC, there are two methods for determining RCP, using a TLC method in conjunction with an rTLC scanner, or a cut and assay technique (see below). The cut and assay method requires the sample to be spotted at the 1 cm line (above the bottom of the plate), and to develop the plate a distance of 10 cm. The plate is then cut at the 6 cm mark (5 cm above the 1 cm demarcation). The percent incorporation (or radiochemical purity, RCP) is calculated by taking the activity counts in the top piece divided by the sum of activity counts in the top and bottom pieces multiplied by 100.

### Quality control of [^68^Ga]Ga-PSMA-11 using either Locametz or Illuccix

QC of Locametz and Illuccix requires the same disposable and non-disposable items listed in Table S4 (see Supplementary Information). It is important to note that for Locametz or Illuccix preparation, the required QC procedures are the same, but there are some differences in the QC release criteria (Table 1). Using tongs take a weight measurement and activity calibration of the dose vial, then with a 1 mL syringe connected to a 1.2 × 38 mm (18Gx1½”) silicone coated needle, insert into the dose vial and pull up ~ 0.1 mL for use in QC analysis. Ensure the pulling of activity is unidirectional, and the solution in the 1 mL syringe is not pushed back into the vial.

**Table 1:** A comparison of the passing quality control criteria for PSMA-11 using Locametz or Illuccix kits

Specifications	Locametz	Illuccix
Maximum Allowed Activity	2.59 GBq	1.85 GBq
Shelf-life (after labeling)	6 h	4 h
Target Volume	5.0 mL (can be diluted to 10 mL with normal saline)	7.5 mL
Radiochemical purity by rTLC	≥ 95%	
Impurity by rTLC	≤ 5%	
Retention factor (Rf)	0.8 – 1.0	0.6 – 1.0
pH	3.2 – 6.5	4.0 – 5.0
Appearance and Visual Inspection	Clear, colorless, and free from particulates	Clear, colorless to slight yellow, and free from particulates
Filter Integrity Test [Cathivex®-GV]	> 344.7 kPa (50 psi)	
Bacterial Endotoxin	< 35 EU/mL	< 23.3 EU/mL
^68^Ge breakthrough (Radionuclidic purity)	≤ 0.001%	

To analyze radiochemical purity by rTLC, using a 1–5 µL pipette, spot a 1 μL aliquot 1 cm above the bottom of a 7 cm TLC plate and place the plate into a TLC chamber with 6 mL of 1:1 (1 M) ammonium acetate solution (aqueous): methanol and cover the chamber (see Supplementary Information for details on preparation of this eluent). Develop the plate in the solution until the solvent front is 1 cm away from the top of the plate which will take about 8–12 min. Since the development time is quite slow, an optional 2nd TLC plate can be developed concurrently to mitigate delays in the event of an invalid TLC test. Dry the developed plate on a hot plate to remove excess water and place the plate on a rTLC plate reader. Follow the package insert directions for passing criteria; briefly, for Locametz, a rTLC purity of ≥ 95%, and a Rf of 0.8–1.0, and for Illuccix, a rTLC purity of ≥ 95%, and a Rf of 0.6–1.0 (Novartis Pharmaceuticals Corporation 2022; Telix Pharmaceuticals 2023).

To analyze for pH, using a 10–50 µL pipette with a pipette tip, a 17.5 µL aliquot is spotted onto a MQuant colorimetric pH indicator strip, the package insert directions for passing criteria are followed: for Locametz, a pH of between 3.2–6.5 is required, and for Illuccix, a pH of between 4.0–5.0. For visual inspection, the dose vial (and/or QC aliquot (~ 0.1 mL) depending on local practices) are inspected visually for color and particulates, and following the package insert directions for passing criteria: for Locametz, the vial should be clear, colorless, and free from particulates, and for Illuccix, the vial should be clear, colorless to slight yellow, and free from particulates (Novartis Pharmaceuticals Corporation 2022; Telix Pharmaceuticals 2023).

To ensure that doses are sterile and free of bacterial endotoxins, three tests are conducted. First, the final product filter used to filter the [^68^Ga]GaCl_3_ into the final kit vial is tested for integrity. The filter is connected to an air line with a pressure regulator and placed into a jar containing Milli-Q water. The pressure regulator is slowly turned up to allow for increased pressure of air to the filter. When the filter is ruptured, determined by flow of air bubbles into the jar, this value is the filter integrity value (bubble point) which should be > 344.7 kPa (50 psi) for the Cathivex®-GV filter, or the manufacturer’s specified pressure if a different filter is used.

Second, bacterial endotoxins testing is performed using the Endosafe endotoxin PTS system. While not required by the Locametz or Illuccix package insert, we include endotoxin testing here as it is listed in European Pharmacopoeia monograph 3044: Gallium (^68^Ga) PSMA-11 Injection (European Pharmacopoeia 2021), and it was straightforward to include as we have historically conducted it when manufacturing [^68^Ga]Ga-PSMA-11 under an FDA-approved Investigational New Drug (IND) application (Rodnick et al. 2020, 2022). Using a 10–50 µL pipette with a pipette tip, 17.5 µL of the dose is added into a 5 mL falcon tube containing 3.9 mL of pre-measured LAL water. The sample is capped and vortexed for 5 s. Using a 1:300 dilution on the Endosafe endotoxin PTS cartridge reader with an Endosafe PTS cartridge, a 25 μL aliquot of the mixed LAL water is added to each of the four channels in the cartridge. The endotoxin test will take 650–800 s to complete. Following USP < 85 > , the maximum allowable endotoxin limit is 175 endotoxin units (EU) per the maximum volume injected (United States Pharmacopeia, 2024a). Thus for Locametz the limit is 35 EU/mL, and for Illuccix the limit is 23.3 EU/mL.

Lastly, sterility testing is performed. Due to the nature of PET radiopharmaceuticals, and in accordance with standard practice when working with PET drugs, the test is completed after the dose is released to the clinic for use and is administered to the patient. While not required by the Locametz or Illuccix package insert, we include sterility testing here as it is also listed in European Pharmacopoeia monograph for [^68^Ga]Ga-PSMA-11 (European Pharmacopoeia 2021), and it was straightforward to include as we routinely conducted sterility testing when manufacturing [^68^Ga]Ga-PSMA-11 under an FDA-approved Investigational New Drug (IND) application (Rodnick et al. 2020, 2022). To determine if bacterial contamination has occurred, tubes of tryptic soy broth (TSB) and fluid thioglycolate medium (FTM) are each inoculated with 250 μL of the dose within 30 h of production. The TSB is incubated at room temperature (20–25 °C) and the FTM is incubated at 30–35 °C for 14 days during and, on days 5, 7, and 14 tubes are visually inspected for microbial growth. Following USP < 71 > , the samples should be clear with no turbidity after 14 days (United States Pharmacopeia, 2024b).

To ensure that the E&Z generators are not leaching long-lived germanium-68 (^68^Ge, half-life 271 days) into batches of [^68^Ga]Ga-PSMA-11, once a week the generators must be checked for germanium breakthrough by testing radionuclidic purity (RNP). This test is required by the Illuccix package insert (Telix Pharmaceuticals 2023) as well as the European Pharmacopeia (Nelson et al. 2022) and US Nuclear Regulatory Commision (Tapp, 2016). While the Locametz package insert does not explicitly require breakthrough testing, it does state that *instructions for use provided by the germanium-68/gallium-68 generator manufacturer should also be followed* (Novartis Pharmaceuticals Corporation 2022). ^68^Ge breakthrough testing is accomplished by pushing 5 mL of 0.1 M ultrapure sterile hydrochloric acid through the generator into a shielded, vented 10 mL vial and transferring a 3 μL aliquot of the eluate into an HPLC vial (or equivalent small vial). The samples are read in a multi-channel analyzer (MCA) with a zoom window of 8–2048 keV for 20 min. The samples are read twice, with the first reading within 12 h after elution and the second reading between 24–120 h after elution. ^68^Ge breakthrough is calculated by using the following equations:

$$1\text{st scan Decay Corrected Counts}=\text{Net peak area}*{1200}^{\left(\text{ln}\left(2\right)*\frac{\text{time after elution in minutes}}{68}\right)}$$

$$2\text{nd scan Decay Corrected Counts}=\text{Net peak area}*{1200}^{\left(\text{ln}\left(2\right)*\frac{\text{time after elution in days}}{271}\right)}$$

$$\text{Percent breakthrough}=100*((2\text{nd scan decay corrected counts})/(1\text{st scan decay corrected counts}))$$

Following the E&Z GalliaPharm® ^68^Ge/^68^Ga generator product description, Nuclear Regulatory Committee and European Pharmacopeia requirements, and the Illuccix package insert, breakthrough values of ^68^Ge should be less than 0.001% of the total radioactivity (Tapp, 2016; Eckert and Zigler, 2021; Nelson et al. 2022; Novartis Pharmaceuticals Corporation 2022; Telix Pharmaceuticals 2023).

### Statistical analysis

Data is expressed as mean ± standard deviation, unless otherwise specified, and statistical analysis is conducted using Prism Version 9 software (GraphPad) with significance tested by comparing the 95% confidence interval (*P* < 0.05). The two main groups are production using Locametz kits, and production using Illuccix kits. The Locametz group is then further subdivided into production using 1.85 GBq E&Z or 3.7 GBq E&Z generators, either using regular needles (RN, 0.7 × 38 mm, 22Gx1½”) or non-metal leaching silicone coated needles (NMLN, 0.6 × 60 mm, 23Gx2 3/8”). The Illuccix group is subdivided into production using 1.85 GBq E&Z generators or a decayed 3.7 GBq E&Z generator (producing less than 1.85 GBq), using either a syringe pump or manual elution, or using the two different sizes of NMLN silicone coated needles (0.6 × 60 mm (23Gx2 3/8”) or 1.2 × 38 mm (18Gx1 1/2”)).

## Results

Using E&Z ^68^Ge/^68^Ga generators at our facility to produce [^68^Ga]Ga-PSMA-11 for clinical use, we prepared Locametz kits (n = 181) from 2nd June 2022 to 9th February 2023, and prepared Illuccix kits (n = 256) from 17th October 2022 to 13th September 2023.

### Locametz production

**Fig. 2:**
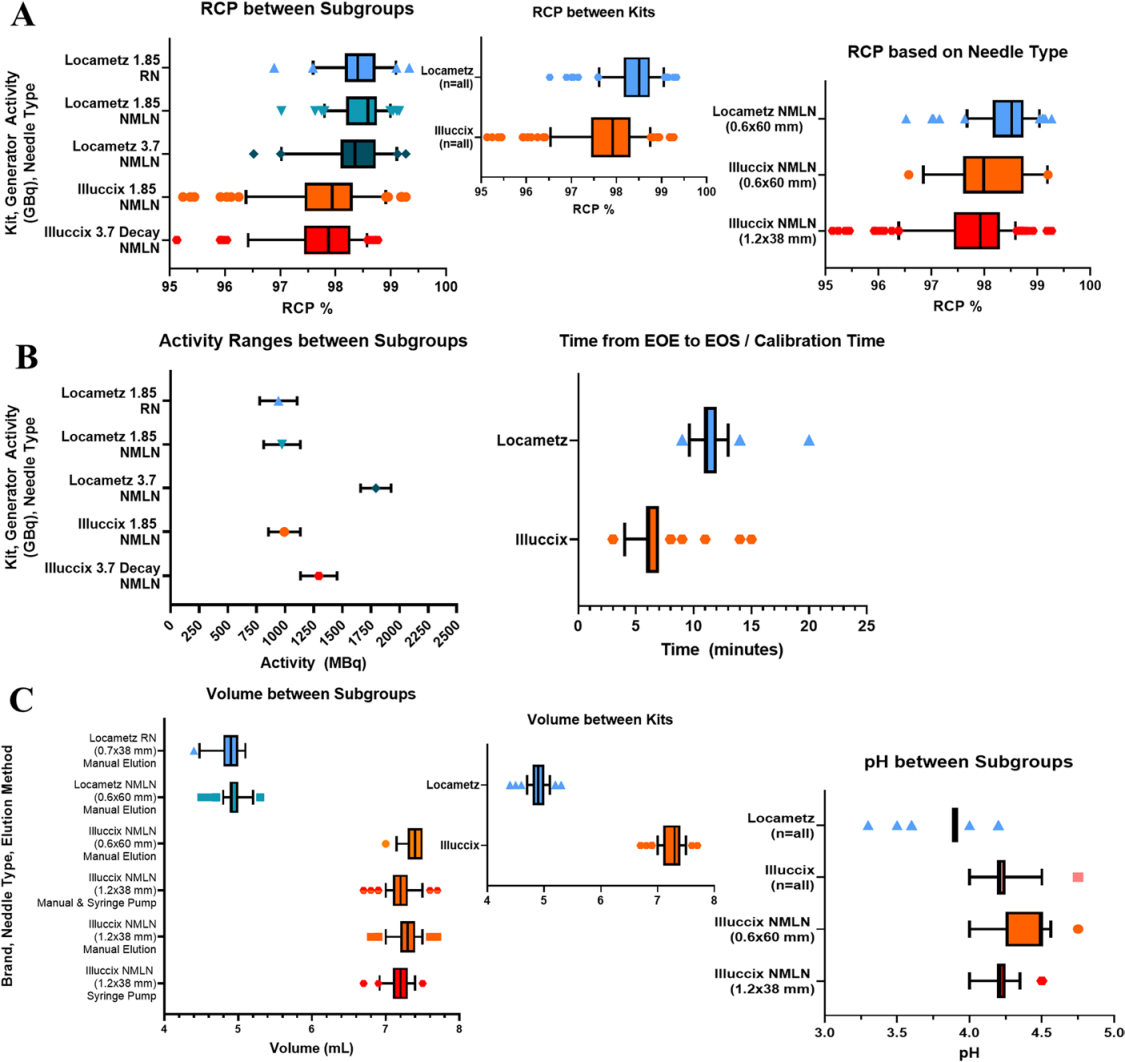
Statistical Analysis of RCP, activity, volume, and pH for the production of Locametz and Illuccix kits and their subgroups analysis. Displayed bars represents the 5–95% percentile of data except for “Activity Ranges between subgroups” which shows the mean ± standard deviation. Symbols are outliers. The radiochemical purity (RCP) by subgroups (generator size, needle type used), by kit (Locametz, Illuccix), and specific type of non-metal leaching needle (NMLN) (**A**). The activity ranges of the dose (at calibration) by subgroup (generator size, needle type used), and the time from end-of-elution (EOE) to end-of-synthesis (EOS) or calibration time (**B**). The volume ranges of the dose by subgroup (generator size, needle type used, and elution method), by kit (Locametz, Illuccix), and the subsequent pH of the subgroups (kit type, needle type) (**C**). Activities are not decay corrected and are compared at time of calibration. RCP were obtained using rTLC and the screening method using a plate reader. Doses that failed were not included (8 for Locametz, 2 for Illuccix) in this analysis. Analysis and figures generated using Prism Version 9 software (GraphPad)

Using the Locametz kits, the overall production success rate was 95.03% (172/181). For the doses that met (or exceeded) the established QC criteria (Table 1) and were released to the clinic for use in patients, the mean RCP by rTLC was 98.41 ± 0.47% (95% CI: 98.34–98.48) (Fig. 2a). The activity at calibration ranged from 0.53 to 2.15 GBq across 5 different lots of 1.85 GBq E&Z generators and 1 lot of 3.7 GBq E&Z generator, with a mean time from end of elution to calibration of 11.28 ± 1.25 min (Fig. 2b). The mean dose volume was 4.93 ± 0.14 mL and a mean pH of 3.9 (range 3.3–4.2) (Fig. 2c). The time of synthesis was approximately 12 min. Including completion of the required paperwork and QC testing, the total time per production was approximately 35 min.

**Fig. 3:**
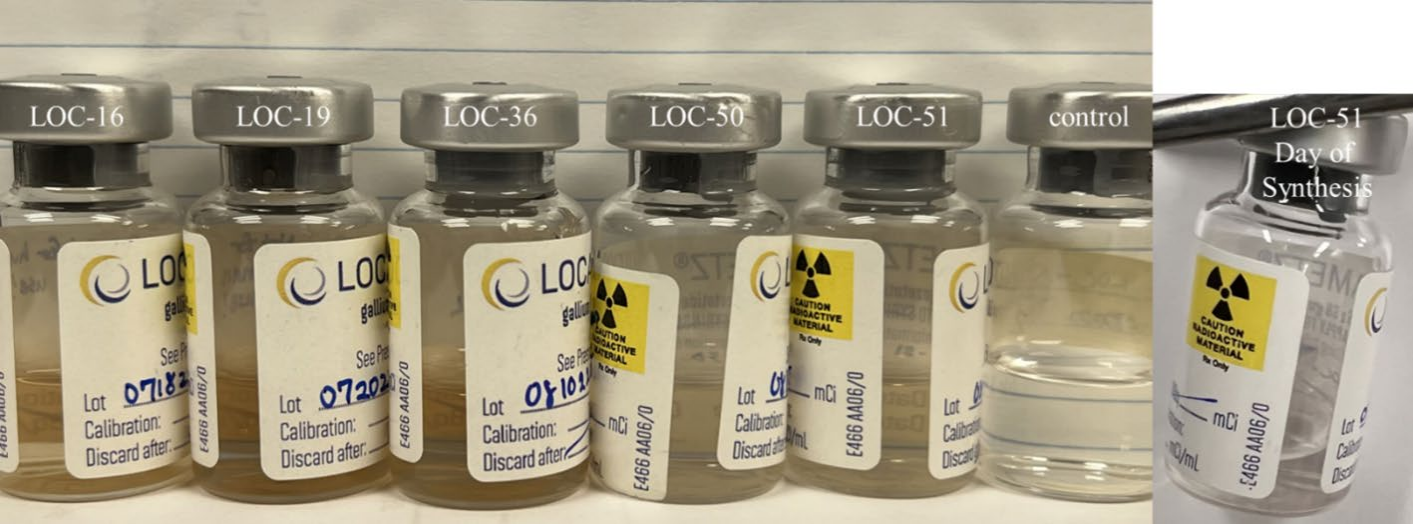
Failed Locametz doses due to discoloration. From Left to Right: Locametz (LOC) lot 16 with 77.97% RCP, lot 19 with 83.16% RCP, lot 36 with 45.71% RCP, lot 50 with 98.8% RCP, lot 51 with 95.36% RCP, and control test using silicone coated non-metal leaching needles with 97.64% RCP. Image taken 1.5 years after synthesis and as a comparison lot 51 image taken day of synthesis (30 min after labeling). Doses with low RCP at end of synthesis had considerably greater color change after one year compared to doses that were passing but had minor discoloration. Comparing discoloration of lot 51 on the day of synthesis and control shows potential issues with using metal needles that are not coated in silicone

For the subset of production using only non-silicone coated needles (RN, 0.7 × 38 mm, 22Gx1½”) for which generator produced [^68^Ga]GaCl_3_ was eluted into the Locametz vial (n = 52), the overall success rate was 84.62% (44/52). For the doses that passed and were released for use in patients, the mean RCP by rTLC was 98.38 ± 0.47% (95% CI: 98.24–98.53) (Fig. 2a). The doses that used non-silicone coated RN needles, led to 8 failures which were not released for clinical use due to < 95% RCP, and/or minor discoloration (pink tinge) of the formulated dose approximately 15–20 min after the addition of [^68^Ga]GaCl_3_ (Fig. 3). Five of the failed batches had RCP < 95% (ranging from 45.71–91.55%), and the remaining 3 vials had passing RCP (ranging from 95.36–98.80%). All other QC parameters passed for these 8 batches. Vials with lower RCP appeared to discolor to a greater extent when re-examined after 1 year. In attempts to recreate the discoloration and identify the likely source for contamination, a variation of shaking and inverting, or not shaking and inverting, “heating” at 27 °C for 7 min or labeling at room temperature for 10 min, and the use of RN needles or silicone coated NMLN needles were tested. Ultimately, swapping the RN needles out for silicone coated NMLN needles led to doses that were clear, colorless, and free of particulates. All other variations (shaking and/or “heating” at 27 °C) using silicone coated NMLN led to passing QC parameters with no color changes. We hypothesize that the discoloration of failed batches was due to the leaching of metal ions from RN needles that could both compete with ^68^Ga during chelation and oxidize the gentisic acid stabilizer that is part of the Locametz formulation. Silicone coated NMLN needles do not leach metal ions and were thus adopted for [^68^Ga]Ga-PSMA-11 kit preparations going forward.

For the subset of productions (n = 129) using silicone coated needles (NMLN, 0.6 × 60 mm, 23Gx2 3/8”), the overall production success rate was 99.22% (128/129). For the doses that passed, the mean RCP by rTLC was 98.42 ± 0.47% (95% CI: 98.33–98.50) (Fig. 2a). For these doses, only 1 failure was observed which was due to the lack of vial inversion and shaking leading to RCP of 91.55%. This issue was rectified by inverting and shaking the vial.

Prior to the most recent package insert for Locametz, which changed the maximum allowed activity from 1.85 GBq to 2.59 GBq, we independently tested the compatibility of “high activity” with Locametz kits using a fractionation approach with a 3.7 GBq and a 1.85 GBq E&Z generator. Using the highest fractions of a 3.7 GBq E&Z generator corresponding to fractions 2 through 4 (the 2nd through 4th mL of 0.1 M HCl used to elute the generator) of the elution, combined with fractions 2 and 3 (the 2nd and 3rd mL of 0.1 M HCl) of an 1.85 GBq E&Z generator, we were able to add 3.16 GBq in 5 mL of 0.1 M HCl into the Locametz vial which, at calibration (11 min later), gave 2.9 GBq. The initial RCP was 97.22% (n = 2) and at 4 h the RCP was 98.96% with all other QC parameters passing (see Supplementary Information, Figure S1ab). The RCP appears higher at 4 h when minor impurities decay below the rTLC limit of detection. A second attempt without fractionation was completed by eluting a 3.7 GBq generator with 6.2 mL of 0.1 M HCl through a 0.22 μm Cathivex-GV filter to give 2.22 GBq at calibration time, with RCP of 98.35% at calibration and 99.23% at 4 h (see Supplementary Information, Figure S2ab). With the established “higher activity” Locametz, subdividing the group using NMLN needles (n = 129) into production using a 1.85 GBq generator (n = 81) and a 3.7 GBq generator (n = 48) showed no differences in product quality and no differences in failure rate. The RCP by rTLC were 98.49 ± 0.39% (95% CI: 98.40 – 98.58) and 98.29 ± 0.57% (95% CI: 98.13–98.46), respectively (Fig. 2a).

### Illuccix production

Using Illuccix kits (n = 256), the overall production success rate was 99.22% (254/256). For the doses that met (or exceeded) established QC criteria (Table 1) and were released for use in patients, the mean RCP by rTLC was 97.81 ± 0.71% (95% CI: 97.73–97.90) (Fig. 2a). The activity at calibration ranged from 0.611 to 1.79 GBq across 6 different lots of 1.85 GBq E&Z generators, and 1 lot of a decayed 3.7 GBq E&Z generator producing less than 1.85 GBq, with a mean time from end of elution to calibration of 6.19 ± 1.17 min (Fig. 2b). To be able to use the decayed 3.7 GBq generator, it took 24 weeks of tri-weekly elution (and ^68^Ge decay) to get to an activity below the 1.85 GBq threshold for use with Illuccix kits. The mean dose volume was 7.25 ± 0.163 mL and the mean pH 4.24 (range 4.0–4.75) (Fig. 2c). The time of synthesis was approx. 8 min. Including completion of required paperwork and QC testing, the total time per production was approximately 30 min.

For the subset of productions (n = 34) using silicone coated needles (NMLN, 0.6 × 60 mm, 23Gx2 3/8”) to transfer the acetate buffer from vial 2 to vial 1 and the elution of [^68^Ga]GaCl_3_ into vial 1, the production success rate was 100% (34/34). These doses had a mean RCP by rTLC of 98.04 ± 0.70% (95% CI: 97.80–98.29) and a mean volume of 7.37 ± 0.12 mL (95% CI: 7.33–7.41). These doses had a mean pH of 4.38 ± 0.17 (95% CI 4.325–4.440) (Fig. 2a,c).

For Illuccix kits (n = 222) prepared using silicone coated needles (NMLN, 1.2 × 38 mm, 18Gx1½”), the production success rate was 99.1% (220/222). For the doses that passed QC and were released for clinical use, the mean RCP by rTLC was 97.78 ± 0.70% (95% CI: 97.68–97.87) and a mean volume of 7.23 ± 0.16 mL (95% CI: 7.21–7.25). These doses had a mean pH of 4.21 ± 0.10 (95% CI: 4.199–4.225) (Fig. 2a,c). In this subset of doses, there were three failures due to low pH (< 2.5), which we attribute to insufficient mixing of the kit components. After vigorously shaking the vial and leaving at room temperature for an additional 5 min, the doses were recalibrated, and a second aliquot was obtained for QC. All three doses had pH within normal range (4.2 and 4.4) and all other QC tests met (or exceeded) release criteria, allowing doses to be released for clinical use.

Among all the Illuccix doses prepared (n = 256), the subset of doses using a 1.85 GBq generator (n = 157) had a success rate of 98.8% (155/157) with mean RCP by rTLC of 97.83 ± 0.73% (95% CI: 97.72–97.94) (Fig. 2a). The doses using a decayed 3.7 GBq generator producing < 1.85 GBq (n = 99) were also used, which had a production success rate of 100% (99/99) and mean RCP by rTLC of 97.74 ± 0.68% (95% CI: 97.61–97.88) (Fig. 2a). There were no major differences between the use of the different generators.

Of all the Illuccix doses (n = 256), the subset of doses using a syringe pump to elute the generator by pushing 6.2 mL of 0.1 M HCl at 2 mL/min had a success rate of 96.9% (63/65), with mean RCP by rTLC of 97.92 ± 0.60% (95% CI: 97.77–98.07) and a mean volume of 7.20 ± 0.17 mL (95% CI: 7.15–7.24) (Fig. 2c). The subset of doses manually eluted by pushing 6.2 mL of 0.1 M HCl at approx. 2 mL/min had a success rate of 100% (191/191) with mean RCP by rTLC of 97.77 ± 0.74% (95% CI: 97.67–97.87) and a mean volume of 7.26 ± 0.16 mL (95% CI: 7.24 – 7.29) (Fig. 2c). Required weekly, breakthrough test was completed on all generators used. The breakthrough was below the limit of detection, which was determined to be < 0.0004% for all generators, meeting the established criteria.

## Discussion

The time required to complete one synthesis of Locametz is around 12 min excluding QC testing (QC time is ~ 23 min); whereas for Illuccix it is around 8 min excluding QC testing (QC time is ~ 22 min). The success rates of production using Locametz and Illuccix kits were comparable, and no significant differences were observed. More importantly, when looking at the subset of productions using silicone coated needles (NMLN) for elution, the rates are the same (1 failure every 128 runs). This suggests that from a production standpoint both Locametz and Illuccix kits have very similar reliability, which makes the decision between which kit to use for routine production of [^68^Ga]Ga-PSMA-11 more nuanced. Comparing our workflows, we assemble our final product vials in an ISO 5 PEC, following aseptic technique, and elute the generators in an ISO 8 hot cell (according to guidelines in USP < 825 >). When using Illuccix kits, we add the acetate vial (vial 2) into the PSMA-11 precursor vial (vial 1) in the ISO 5 PEC, prior to the addition of the [^68^Ga]GaCl_3_ from the generator in the ISO 8 hot-cell. This is a deviation from the package insert, which recommends adding the acetate and PSMA-11 solution into the [^68^Ga]GaCl_3_. This order of events would require us to bring the eluted [^68^Ga]GaCl_3_ back to the ISO 5 PEC to add the acetate and PSMA-11 solution. This process becomes unnecessarily complicated, with higher radiation exposure rates to production staff, and therefore has not been adopted for routine production. This minor change to the process order has had no impact upon product quality and is considered validated after 254 successful productions. For Locametz, this is not an issue because a separate buffer (or pH adjusting) solution is not required.

Excluding the eight failures related to using non-silicone coated needles (RN), we attribute the remaining failures (1 for Locametz, and 1 for Illuccix) to improper mixing. The Locametz package insert suggests the inversion of the dose vial to help with incorporation and mixing of the lyophilized buffer, but no agitation. Improper mixing of the Locametz vial led to a failure, which suggests that agitation of the vial increases the consistency of labeling by adequately mixing the buffer agent and HCl. Although the package insert suggests that labeling is complete within 5 min, it also recommends the vials be labeled at temperatures within 20 °C to 30 °C, which in colder climates may not always be possible (Novartis Pharmaceuticals Corporation 2022). Extending the labeling time to 10 min further ensures consistency without the need to “heat” the reaction above room temperature (e.g. to 27 °C) to be within the allowed temperature range.

When labeling NetSpot ([^68^Ga]Ga-DOTATATE), agitation of the vial is not recommended due to potential leaching of competing metals such as zinc from the vial septa leading to poor incorporation of ^68^Ga (III), as Zn(II) can also form stable complexes with DOTA (Fang et al. 2020; Advanced Accelerator Applications USA Inc., 2021). The Locametz package insert recommends inversion of the vial to help with mixing but recommends against agitation or stirring (Novartis Pharmaceuticals Corporation 2022). However, the affinity of HBED-CC for Ga(III) is higher than the affinity between HBED-CC and Zn(II) (Ma et al. 1994; Iudicello et al. 2021) and so we believe competing chelation of Zn is less likely to be an issue when preparing [^68^Ga]Ga-PSMA-11 and this has been borne out in our experience thus far.

For Illuccix, the package insert does not specify the need to shake the vial after the addition of [^68^Ga]GaCl_3_ to the acetate buffered ligand solution (Telix Pharmaceuticals 2023). However, with our production runs, we saw 3 instances where the lack of vial agitation led to poor incorporation of ^68^Ga due to incorrect reaction pH (< 2.5). Our first low pH dose led to a failure, as we did not shake the vial on that occasion; however, with our second and third cases, the vial was shaken. In each case the vials were shaken again to ensure proper mixing of the [^68^Ga]GaCl_3_ and the acetate buffered ligand solution, and this led to appropriate incorporation (RCP ≥ 95%) and pH of ~ 4.2. Although the incorrect pH only contributed to 1.17% (3/256) of all Illuccix production runs, shaking the vial increases the consistency for efficient labeling, and is therefore recommended.

When Locametz production was conducted using non-silicone coated needles (RN, 0.7X38 mm, 22Gx1½”), several failures resulted. These were due to low incorporation of ^68^Ga and/or discoloration of the product. We have not used Illuccix kits with these non-silicone coated needles, but the failed Locametz productions using these needles suggest that using silicone coated needles (NMLN) improves labeling and so they are also recommended for use with Illuccix. Although both Locametz and Illuccix kits contain the same “precursor”, the excipients and formulation of the kits are different (see Supplementary Information, Table S1). The QC criteria for appearance with Locametz requires “clear, colorless, particulate free” solutions (Novartis Pharmaceuticals Corporation 2022), whereas Illuccix allows for “clear, colorless to slight yellow, particulate free” solutions (Telix Pharmaceuticals 2023). The difference in QC appearance criteria is attributed to the differences in formulation. When looking at the components used to prevent radiolysis of PSMA-11, for Locametz, 1 mg of gentisic acid (radioprotectant) is used, while for Illuccix, 10 μg of **D**-mannose (a reducing sugar, acting as a radioprotectant) is used.

The color change observed in Locametz kits when using non-silicone coated needles noted above could result from complexation of metals leaching from such needles, and/or oxidation of gentisic acid, which is known to give a faint pinkish, yellowish color (Hosokawa et al. 2020). The purpose of gentisic acid in the dose vial is to prevent the oxidation of PSMA-11, acting as a sacrificial substrate for oxidative chemical reactions meaning it has radioprotective effects in the formulation, but could also be oxidized by the presence of metal ions leaching from non-silicone coated needles. Indeed, by switching to needles that have a low potential for leaching metals capable of catalyzing such oxidation, the discoloration was no longer observed, and therefore it is recommended that silicone coated needles (either 0.6 × 60 mm, 23Gx2 3/8” or 1.2 × 38 mm, 18Gx1 1/2”) be used to produce Locametz. Detailed analysis via HPLC and/or mass spectrometry may reveal whether the color change is due to one pathway or the other (e.g. by confirming the presence of other metal-PSMA complexes) and will be the subject of future studies. It is also suggested that the recommendation to use silicone coated needles be included in a future version of the package Insert as it appears critical to the successful preparation of Locametz kits.

The silicone coated needles (NMLN) used to label Locametz and Illuccix kits were from B. Braun (brand name Sterican®). We routinely use these needles with NetSpot kits to produce [^68^Ga]Ga-DOTATATE) and thus we also selected them to label PSMA-11 and make our entire ^68^Ga -labeling workflow more uniform. Originally, the 0.6 × 60 mm (23Gx2 3/8”) needles were used, which provided sufficient length to pull up all the acetate buffer from Illuccix vial 2 without needing to invert the vial. Due to regulatory changes preventing us from obtaining these needles from a third-party, a new length of needles was used (1.2 × 38 mm, 18Gx1 1/2”). Since these needles were shorter, and the bore larger, it made it very difficult to pull up all 2.5 mL of the acetate buffer from vial 2. The acetate buffer in vial 2 needed to be inverted, and because the needle was no longer as flexible as the 0.6 × 60 mm needles, required more manipulations to acquire an “accurate” volume. Ultimately, this change in needles led to minor differences in the final dose volume. Even with these volume changes, which presumably were due to the differences in acetate buffer added and not the HCl eluted through the generator, there were no significant differences in RCP or pH of the labeled product. This suggests that although the addition of 2.5 mL of acetate solution (vial 2) is recommended by the manufacturer in the package insert, a volume of ~ 2.2 mL is still tolerated by the kit. This is further demonstrated by the fact that the success rate was not significantly altered between the two sizes of needles (100% vs. 99.1%). If we exclude the 2 failures which were unrelated to the volume of acetate added, the success rate was 100% (failures were due to inadequate mixing and user error in operating the syringe pump). Although there are no differences in QC results between the two silicone coated needles, when selecting the length and bore size of the needles used for [^68^Ga]GaCl_3_ elution and QC sample draw, we recommend a longer length, smaller bore size needle, as this will make the assembly of the dose vial in an ISO 5 PEC more streamlined with fewer manipulations and hand exposure to radiation for staff.

We demonstrated that adding higher amounts of ^68^Ga to Locametz kits (> 1.85 GBq allowed by the package insert at the time) led to doses that still met all QC criteria at 4 h post-EOS. By using a fractionation approach we were able to demonstrate that up to 3.16 GBq (decay-corrected activity added to the vial = 3.5 GBq) was tolerated. Since a 3.7 GBq E&Z generator is expected to only produce 80% of its maximum activity, successful production using > 2.96 GBq of ^68^Ga allows use a 3.7 GBq generator for producing Locametz throughout the generator’s lifetime. When comparing the Locametz lower activity (< 1.85 GBq) and higher activity (> 1.85 GBq) productions, there were no significant differences in RCP or other QC test results. In marked contrast, when > 1.85 GBq of ^68^Ga were added to an Illuccix kit, rHPLC analysis showed < 95% RCP. In our hands, this did not align with rTLC test results, which showed RCP > 95%. This discrepancy requires further investigation but meant we did not use > 1.85 GBq of ^68^Ga with Illuccix in this work.

Use of a syringe pump to elute the ^68^Ga generator when labeling Illuccix led to similar outcomes as manual elution, and we observed no major changes in RCP, volume, pH or other QC test results between the two approaches. A syringe pump can control the elution rate to a greater extent than manual elution, which tends to be faster than 2 mL/min and offers improved consistency of pressure applied. Since the use of a syringe pump is equivalent to manual elution, we recommend the use of a syringe pump to further decrease radiation hand exposure during generator elution. However, the use of a syringe pump can also increase the risk of user error, which led to one of our failures and careful checking of the setup is recommended. It is important to note that we used 6.2 mL of 0.1 M HCl to elute the generators because we wished to include a 0.22 μm Cathivex-GV filter in-line for terminal sterilization as part of the manufacturing process. The filter traps around 1.2 mL of solution (dead volume) which is lost and therefore accounted for by pushing 1.2 mL more HCl through the generator. Since the generator elution is not homogenous, minimal activity is lost on the filter using this larger volume of HCl (Roesch and Riss, 2012), and we showed it does not impact the volume of the final product; we obtained a final volume for Illuccix of 7.2 ± 0.2 mL (package insert states 7.5 mL) and a final volume for Locametz of 4.9 ± 0.1 mL (package insert states ≤ 10 mL). In a radiopharmacy or nuclear medicine department in an ISO 5 PEC in a clean room (ISO 7 secondary engineering control (SEC)), 5 mL of 0.1 M HCl can be used with or without a filter in-line into the vial. Whichever approach is chosen, a site should validate that inclusion of a sterilizing filter does not impact product quality.

There are two methods for determining RCP using TLC, an iTLC method requiring the use of a radioTLC scanner / plate reader to scan the plate, and a “cut and count” technique. The European Pharmacopeia also allows for use of radio-HPLC to determine RCP of [^68^Ga]Ga-PSMA-11 (European Pharmacopeia, 2021), although that was not included in the present work. Both the scanner and cut and count TLC methods are interchangeable, and therefore both methods can be used to determine RCP. Currently the Locametz package insert only specifies using the scanning method to analyze the TLC plates. On the other hand, the Illuccix package insert allows for either method of analysis. Since the “precursors” are the same, and the synthesis processes are comparable (free ^68^Ga and [^68^Ga]colloids will stay at the origin, and [^68^Ga]Ga-PSMA-11 will move to the solvent front), both kits give similar results and thus we posit that either method can also be used for Locametz as well. For this work, we used the radioTLC scanner method employing an Eckert and Ziegler AR-2000 TLC scanner and 1 µL of sample. The Illuccix package insert states to use one drop while the European Pharmacopeia recommends 5 µL, and sites should develop (and validate) a method that is compatible with their TLC equipment (notably, using 5 µL of sample might be appropriate if using the cutting technique to have more activity and ensure sufficient counts in the dose calibrator). We also found that drying the TLC plates before scanning increases consistency of the radioTLC. Since the solvent is aqueous/methanol based, evaporation of the solvent usually takes time, so we dry the plates on a Corning PC-200 lab hotplate (set to 8/10, 450 °C) for approx. 10–15 s, until evaporation of the TLC solvent is visually apparent.

We conducted sterility and endotoxin testing for the products made in this work. While not required by the Locametz or Illuccix package inserts, both tests are listed in European Pharmacopoeia monograph 3044: Gallium (^68^Ga) PSMA-11 Injection (European Pharmacopoeia 2021), and were straightforward to include because we have historically conducted such tests when manufacturing [^68^Ga]Ga-PSMA-11 under an FDA-approved Investigational New Drug (IND) application (Rodnick et al. 2020, 2022). They allowed us to gather data around routine manufacture of [^68^Ga]Ga-PSMA-11 using the Locametz and Illuccix kits, demonstrating that both generate product that is consistently sterile and free of endotoxins. If a given site is required to meet the package insert, but not the European Pharmacopeia, these tests could be eliminated and replaced with media fill testing to validate aseptic technique (cf. compounding of ^99m^Tc-labeled radiopharmaceuticals) (US Food and Drug Administration 2008; Vincenti et al. 2022).

**Fig. 4:**
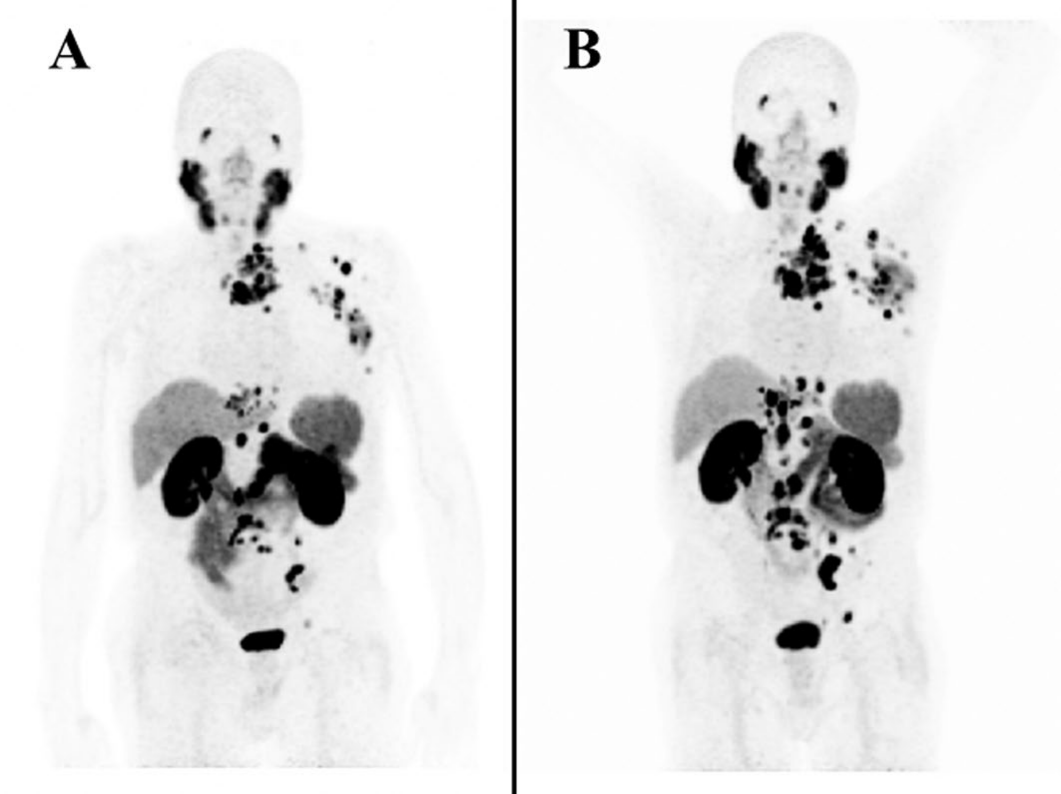
[^68^Ga]Ga-PSMA-11 PET Scan using Locametz and Illuccix Kits. [^68^Ga]Ga-PSMA-11 PET scan first using Locametz (**A**) then Illuccix (**B**) 112 days apart demonstrating avid metastatic disease in abdominal pelvic lymph nodes, thoracic and supraclavicular lymph nodes and bones. Patient was not on active treatment during the interval between scans. Image courtesy of Dr. Ka Kit Wong

A summary of recommendations for the labeling of PSMA-11 using Locametz or Illuccix kits is presented in Table 2. From a clinical perspective, PSMA-11 scans using Locametz or Illuccix kits are functionally the same. Figure 4 shows scans from a patient who received a [^68^Ga]Ga-PSMA-11 PET scan using Locametz first, then a second [^68^Ga]Ga-PSMA-11 PET scan using Illuccix 112 days after the first scan. The patient was not on treatment between scans, and was only taking dexamethasone. In both images, the patient has avid metastatic disease in the abdominal pelvic lymph nodes, thoracic and supraclavicular lymph nodes, and bones. These images, from the same patient, demonstrate the similarities of scans acquired using [^68^Ga]Ga-PSMA-11 prepared using each kit.

**Table 2:** A summary of recommendations for labeling PSMA-11 using Locametz or Illuccix kits

	Locametz kit	Illuccix kit
Generator produced [^68^Ga ]GaCl_3_	Can use E&Z, IRE EliT Galli EO, or ITM GeGant generators	Can use E&Z with *configuration A* and IRE EliT Galli EO with *configuration B*
Cyclotron produced [^68^Ga ]GaCl_3_	*Not allowed, not recommended*	Following package insert directions using GE GaCl_3_ cassette with FASTLab synthesizer. Disregard our changes to the order of acetate solution addition
Activity at calibration and expiration	Up to 3.16 GBq for up to 4 h or up to 2.59 GBq for up to 6 h	Up to 1.85 GBq for up to 4 h
Needles	Silicone coated non-metal leaching needles, preferably needles with longer length and smaller bore size	
Addition of buffer solution	*not necessary, one vial formulation*	Add the acetate solution into the ligand vial first then add [^68^Ga ]GaCl_3_. There is approximately 0.3 mL of tolerance built into the amount of acetate solution added
Inversions after the addition of [^68^Ga ]GaCl_3_	2 inversions	no inversions
Shaking the vial after addition of [^68^Ga ]GaCl_3_	Vigorously (up, down, left, right) for 2 s	
Labeling Time at room temperature	10 min	5 min
Quality Control, RCP by rTLC	Either scanning or cutting method	
Use of a syringe pump	Preferred to reduce hand exposure and increase elution rate consistency

The labeling workflow for PSMA-11 with generator produced [^68^Ga]GaCl_3_ using Locametz or Illuccix kits are similar, and clinical images obtained using [^68^Ga]Ga-PSMA-11 prepared with either kit are considered equivalent. This reinforces the notion that selection of a given kit is primarily dependent on production method preferences which are compared in Table 3. The Locametz kit with a 1-vial formulation makes vial assembly easier and eliminates errors due to transfer between vials. Locametz kits allow the addition of more activity (3.16 GBq) when compared to Illuccix (1.85 GBq) and a longer shelf-life after labeling (6 h vs 4 h, respectively). Locametz kits can be used with more generators, including E&Z, IRE EliT Galli EO, and ITM GeGant, whereas the Illuccix kits can only be used with E&Z and IRE Elit Galli EO generators. Illuccix can be used with cyclotron-produced ^68^Ga, whereas this is not included in the Locametz package insert. From a quality control perspective, both kits have similar release criteria, and either the scanning or cutting method for rTLC analysis can be used for both kits. The retention factor (Rf) cut-off for Locametz is tighter (0.8–1.0), whereas the Illuccix Rf cut-off is more accommodating (0.6–1.0), which also needs to be accounted for.

**Table 3:** A comparison of production method and source of [^68^Ga]GaCl_3_ for Locametz and Illuccix Kits

	Locametz kit	Illuccix kit
Use with E&Z Generator	Yes	Yes, Configuration A only
Use with IRE EliT Galli EO Generator	Yes	Yes, Configuration B only
Use with ITM GeGant Generator	Yes	No
Use with Cyclotron Produced [^68^Ga ]GaCl_3_	No	Yes, Configuration A only
Vials in Kit Formulation	1	3
Dilution with normal saline	Yes, to 10 mL	No
Maximum activity at calibration	3.16 GBq	1.85 GBq
Shelf-Life after labeling	6 h	4 h
Time for radiolabeling at room temperature	10 min	5 min
Plate development time due to Rf criteria	Longer time (tighter Rf criteria)	Shorter time (looser Rf criteria)

Finally, for facilities who are considering using either or both kits, due to cost, workflow or other reasons, the transition between kits is close to seamless. A similar list of non-consumable and consumable products are used which makes switching straightforward (see Supplementary Information, Tables S2 through S4). For radiopharmacies and nuclear medicine departments experienced with general nuclear medicine, the assembly and setup for Locametz is similar to the labeling of numerous ^99m^Tc kits (e.g. [^99m^Tc]MDP, [^99m^Tc]DTPA, [^99m^Tc]tetrofosmin (Myoview)) whereas the assembly and setup for Illuccix is closer to the labeling of other ^99m^Tc kits (e.g. [^99m^Tc]bicisate (Neurolite), [^99m^Tc]sulfur colloid without heating), or [^68^Ga]Ga-DOTATATE (NetSpot). For facilities used to more traditional PET drug manufacturing like FDG, the use of Locametz or Illuccix kits may require new workflows and techniques as generator-based production has not traditionally been widely used at such sites, but this can readily be accomplished through implementation of appropriate standard operating procedures. While this report has focused upon use of Locametz and Illuccix kits in conjunction with ^68^Ge/^68^Ga generators, some might also prefer Illuccix kits due to their compatibility with cyclotron-produced ^68^Ga (Telix Pharmaceuticals 2023).

## Conclusions

In conclusion, preparation of [^68^Ga]Ga-PSMA-11 using Locametz or Illuccix kits have comparable workflows and also provide equivalent PSMA PET images. Therefore, excluding cost and contracting considerations, selecting a kit for day-to-day use ultimately depends on the versatility of these kits and the preferred workflow at a given site. Both kits had comparable failure rates in our hands (1 in 128 vials), although we have observed that using silicone coated non-metal leaching needles, as well as vial agitation after the addition of [^68^Ga]GaCl_3,_ leads to improved labeling consistency. Additionally, labeling Locametz kits at room temperature for 10 min (instead of 5 min) also improved consistency, without substantial delay in workflow. When comparing the two kits, traditional radiopharmaceutical facilities familiar with generator elution and a “kit-based approach” may prefer to use Locametz kits due to the similarities with the general ^99m^Tc workflow, whereas PET manufacturing facilities making FDG might prefer Illuccix kits due to the versatility of the gallium-68 source (generator or cyclotron). Overall, the commercial availability of 2 approved kits for production of [^68^Ga]Ga-PSMA-11 PET has facilitated ready access to this important new prostate cancer imaging agent, and underpinned successful introduction of Pluvicto radiotherapy.

## Supplementary Information

Additional file 1.

Additional file 2.

## Abbreviations

FTM: Fluid thioglycolate medium
HPLC: High pressure liquid chromatography
iTLC: Instant thin layer chromatography
LAL: Limulus amoebocyte lysate
MCA: Multi-channel analyzer
NMLN: Non-metal leaching silicone coated needles, either 0.6 × 60 mm (23G 2 3/8”) needles or 1.2X38 mm (18G 1 1/2”) needles
PET: Positron emission tomography
QC: Quality control
RCP: Radiochemical purity
Rf: Retention factor
RN: Non-silicone coated, regular needles, or 0.7X38 mm (22G 1½”) needles
RNP: Radionuclidic purity
rTLC: Radio thin layer chromatography
TSB: Tryptic soy broth

## Declarations

### Availability of data and materials

Provided in the Supplementary Information is a list of Formulation, components of the Locametz and Illuccix kits (Table S1), as well as a list of disposable and non-disposable items used in both the production of Locametz (Table S2) and Illuccix (Table S3). Additionally, required materials for quality control is also provided (Table S4). Provided in an Excel sheet (Appendix A) is the raw data for all 437 PSMA-11 production batches reported and analyzed in this article. The preparer’s names have been replaced with generic names (Person A, Person B, etc.) in the Excel sheet, but reflect the actual radiochemists’s role for each batch.
