# Supplementary material for: A comparison of routine [68Ga]Ga-PSMA-11 preparation using Locametz and Illuccix kits
Source: EJNMMI Radiopharm Chem. 2024 Dec 18;9:87. doi: 10.1186/s41181-024-00317-4 (PMC11655735; doi:10.1186/s41181-024-00317-4)
Supplement: Supplementary file 1 — Additional file 1. [file 41181_2024_317_MOESM1_ESM.docx]

**A Comparison of Routine [^68^Ga]Ga-PSMA-11 Preparation using Locametz and Illuccix Kits**

**Supplementary Information**

Author: Ivan E. Wang^1^, Luke J. Morrissette^2^, Ka Kit Wong^2^, Allen F. Brooks^2^, Marianna Dakanali,^2^ Peter J. H. Scott^1,2,3†^

1. Department of Medicinal Chemistry, College of Pharmacy, University of Michigan, Ann Arbor, MI 48109, USA

2. Department of Radiology University of Michigan, Ann Arbor, MI 48109, USA

3. Department of Pharmacology, University of Michigan, Ann Arbor, MI 48109, USA

**†Corresponding Author:** Peter J. H. Scott

Department of Radiology, University of Michigan, 2276 Medical Science 1, 1301 Catherine St. Ann Arbor, MI 48109-5610, USA. Email: pjhscott@umich.edu

**Contents:**

**Table S1:** Formulation, components of the Locametz and Illuccix kits, and necessary Gallium-68 chloride required to reconstitute kit.

**Table S2:** Disposable and Non-disposable Items Required for the Production of Locametz

**Table S3:** Disposable and Non-disposable Items Required for the Production of Illuccix

**Table S4:** Disposable and Non-disposable Items Required for Quality Control of Locametz or Illuccix

**Preparation of TLC Eluent**

**Figure S1a:** rTLC of Fractionation Approach using Locametz, initial

**Figure S1b:** rTLC of Fractionation Approach using Locametz, 4-hour stability

**Figure S2a:** rTLC of initial 3.7 GBq generator test using Locametz, initial

**Figure S2b:** rTLC of initial 3.7 GBq generator test using Locametz, 4-hour stability

**Table S1**: Formulation, components of the Locametz and Illuccix kits, and necessary Gallium-68 chloride required to reconstitute kit.

|  | Locametz (gozetotide, PSMA-11) | | | | |
| --- | --- | --- | --- | --- | --- |
|  | Component | Use | mass  (μg) | mass (μmol) | volume (mL) |
| **Gallium Generator** | **Gallium-68 chloride** | **Radioisotope** | **0.00441** | **0.0000252** | **-** |
|  | **0.1 M HCl** | **Diluent** | **18230** | **500** | **qs 5.0** |
| Ligand Vial (Vial 1) | Gozetotide (PSMA-11) | Ligand | 25 | 0.02640 | - |
|  | Gentisic acid | Radioprotectant | 1000 | 6.4885 | - |
|  | Sodium acetate trihydrate | Buffer (Acid) | 78000 | 573.2 | - |
|  | Sodium Chloride | Formulation / osmolarity adjustment | 40000 | 684.5 | - |

|  | Illuccix (gozetotide, PSMA-11), *Configuration A* | | | | | |
| --- | --- | --- | --- | --- | --- | --- |
|  | Component | | Use | mass  (μg) | mass  (μmol) | volume (mL) |
| **Gallium**  **Generator** | **Gallium-68 chloride** | | **Radioisotope** | **0.00315** | **0.000018** | **-** |
|  | **0.1 M HCl** | | **Diluent** | **18230** | **500** | **qs 5.0** |
| Vial 1 | Gozetotide (PSMA-11) | | Ligand | 25 | 0.02640 | - |
|  | D-mannose | | Bulking agent / Stabilizer | 10 | 0.05551 | - |
| Vial 2A | Sodium acetate (anhydrous) | | Buffer (Base) | 150000 | 1828.60 | - |
|  | 0.292 M HCl | HCl | Diluent, Buffer (Acid) | 26615.8 | 730 | - |
|  |  | SWFI |  | - | - | qs 2.5 |
| Vial 3 | Sterile Vacuumed Vial | | Intermediate vial | - | - | - |

|  | Illuccix (gozetotide, PSMA-11), *Configuration B* | | | | | |
| --- | --- | --- | --- | --- | --- | --- |
|  | Component | | Use | mass  (μg) | mass (μmol) | volume (mL) |
| **Gallium Generator** | **Gallium-68 chloride** | | **Radioisotope** | **0.00315** | **0.000018** | **-** |
|  | **0.1 M HCl** | | **Diluent** | **4011** | **110** | **qs 1.1** |
| Vial 1 | Gozetotide (PSMA-11) | | Ligand | 25 | 0.02640 | - |
|  | D-mannose | | Bulking agent / Stabilizer | 10 | 0.05551 | - |
| Vial 2B | Sodium acetate (anhydrous) | | Buffer (Base) | 150000 | 1828.60 | - |
|  | 0.175 M HCl | HCl | Diluent, Buffer (Acid) | 40835.2 | 1120 | - |
|  |  | SWFI |  | - | - | qs 6.4 |
| Vial 3 | Sterile Vacuumed Vial | | Intermediate vial | - | - | - |

Generator produced [^68^Ga]GaCl_3_ gives specific activity of 102745 GBq/g. In **bold** are the components of the ^68^Ge/^68^Ga generator eluate which is added/eluted into the dose vial components. The quantity of Gallium-68 chloride is calculated using the maximum allowed activity. The maximum specific activity of [^68^Ga]Ga-PSMA-11 is 64.75 GBq/g for Locametz and 46.25 GBq/g for Illuccix.

**Table S2**: Disposable and Non-disposable Items Required for the Production of Locametz

| **Item** | **Product Description** | **Product Number** | **Quantity** | **Use** |
| --- | --- | --- | --- | --- |
| **Non-disposable Items** | | | | |
| E&Z Germanium-68/Gallium-68 Generator | 1.85 GBq or 3.7 GBq E&Z generator | DMF #28741 | 1 | Source of [^68^Ga]GaCl_3_ for labeling |
| Generator Eluent | 0.1 M Ultrapure Sterile Hydrochloric Acid (HCl) | n/a, must use E&Z provided HCl | 6.2 mL | Solution used to elute generator |
| Lead or Tungsten Shielding | Lead or Tungsten Shielding appropriate for PET isotopes, fitting 10 mL vial | n/a | 2 | Shielding to attenuate ionizing radiation |
| Scale | Small electronic scale capable of measuring from 0.1 g to 100 g | n/a | 1 | Used to measure volume |
| **Disposable Items** | | | | |
| ^PEC^Locametz Kit | Advance Accelerator Applications, Novartis | 69488-017-61 | 1 kit/vial | Formulated Product vial |
| ^PEC^21 G 2” Needle | Becton Dickinson (BD) Needles, sterile, Green Hub | 305129 | 1 | Needle for sterile vent filter |
| ^PEC^Silicone coated 18 G 1½” Needle | B. Braun Medical Sterican® Needles, sterile, 1.20x40 mm short bevel, Pink Hub | 4665120 | 1 | Needle for sterile product filter (for [^68^Ga]GaCl_3_) |
| ^PEC^0.2 μm sterile vent filter | Millex®-FG, 25mm diameter, 0.2 μm hydrophobic PTFE membrane, sterile | SLFG025LS | 1 | Sterile filter for displaced gas to exhaust/vent out of the product vial |
| †^PEC^0.22 μm sterile product filter | Cathivex®-GV, 25mm diameter, 0.22 μm hydrophilic PVDF membrane, sterile | SLGV0250S | 1 | Sterile filter for [^68^Ga]GaCl_3_ into the product vial |
| 10 mL Organic Syringe | Norm-Ject®, 10 mL Luer Lock Tip Syringe, sterile, translucent plunger | NJ-4606728-02 | 1 | Syringe used to pull up generator eluent |

†Items marked with this symbol, are not required if following state board of pharmacy/ package insert rules and regulations and not cGMP under a PET manufacturing facility following 21 CFR 212.

Items with a superscript “PEC” will be wiped with 70% isopropyl alcohol into a primary engineered control (PEC) for aseptic assembly of the final dose vial to be dispensed or sent to a radiopharmacy for subsequent dose drawing.

E&Z – Eckart and Ziegler, QC – quality control

**Table S3**: Disposable and Non-disposable Items Required for the Production of Illuccix

| **Item** | **Product Description** | **Product Number** | **Quantity** | **Use** |
| --- | --- | --- | --- | --- |
| **Non-disposable Items** | | | | |
| E&Z Germanium-68/Gallium-68 Generator | 1.85 GBq generator or decayed 3.7 GBq generator producing less than 1.85 GBq, E&Z generator | DMF #28741 | 1 | Source of [^68^Ga]GaCl_3_ for labeling |
| Generator Eluent | 0.1 M Ultrapure Sterile Hydrochloric Acid (HCl) | n/a, must use E&Z provided HCl | 6.2 mL | Solution used to elute generator |
| Lead or Tungsten Shielding | Lead or Tungsten Shielding appropriate for PET isotopes, fitting 10 mL vial | n/a | 2 | Shielding to attenuate ionizing radiation |
| Scale | Small electronic scale capable of measuring from 0.1 g to 100 g | n/a | 1 | Used to measure volume |
| **Disposable Items** | | | | |
| Illuccix Kit | ^PEC^Telix Pharmaceuticals, Configuration A, Vial 1, Gozetotide vial, blue flip-off cap | 74725-101-25 | 1 vial | Formulated Product vial |
|  | ^PEC^Telix Pharmaceuticals, Configuration A, Vial 2, Acetate Buffer Vial, red flip-off cap | 74725-102-25 | 1 vial, 2.5 mL buffer | Buffer vial required to pH adjust the 0.1 M HCl |
|  | Telix Pharmaceuticals, Configuration A, Vial 3, Sterile Vacuumed Reaction Vial, white flip-off-cap | n/a | 0 | *Not used in procedure, but included in Illuccix Kit* |
| ^PEC^21 G 2” Needle | Becton Dickinson (BD) Needles, sterile, Green Hub | 305129 | 1 | Needle for sterile vent filter |
| ^PEC^Silicone coated 18 G 1½” Needle | B. Braun Medical Sterican® Needles, sterile, 1.20x40 mm short bevel, Pink Hub | 4665120 | 2 | Needle for sterile product filter (for [^68^Ga]GaCl_3_) |
| ^PEC^0.2 μm sterile vent filter | Millex®-FG, 25mm diameter, 0.2 μm hydrophobic PTFE membrane, sterile | SLFG025LS | 1 | Sterile filter for displaced gas to exhaust/vent out of the product vial |
| †^PEC^0.22 μm sterile product filter | Cathivex®-GV, 25mm diameter, 0.22 μm hydrophilic PVDF membrane, sterile | SLGV0250S | 1 | Sterile filter for [^68^Ga]GaCl_3_ into the product vial |
| 10 mL Organic Syringe | Norm-Ject®, 10 mL Luer Lock Tip Syringe, sterile, translucent plunger | NJ-4606728-02 | 1 | Syringe used to pull up generator eluent |
| ^PEC^5 mL Organic Syringe | Norm-Ject®, 5 mL Luer Lock Tip Syringe, sterile, translucent plunger | NJ-4606710-02 | 1 | Syringe used to pull up buffer from Vial 2 |

†Items marked with this symbol, are not required if following state board of pharmacy/ package insert rules and regulations and not cGMP under a PET manufacturing facility following 21 CFR 212.

Items with a superscript “PEC” will be wiped with 70% isopropyl alcohol into a primary engineered control (PEC) for aseptic assembly of the final dose vial to be dispensed or sent to a radiopharmacy for subsequent dose drawing.

E&Z – Eckart and Ziegler, QC – quality control

**Table S4**: Disposable and Non-disposable Items Required for Quality Control of Locametz or Illuccix

| **Item** | **Product Description** | **Product Number** | **Quantity** | **Use** |
| --- | --- | --- | --- | --- |
| **Non-disposable Items** | | | | |
| Lead or Tungsten Shielding | Lead or Tungsten Shielding appropriate for PET isotopes, fitting 3 mL vial | n/a | 1 | Shielding to attenuate ionizing radiation |
| Dose Calibrator | Capintec CRC-15r, calibration code 416 | n/a | 1 | Activity Reading |
| TLC Chamber | Glass chamber or battery jar with lid, minimum 8 cm tall | n/a | 2 | Used to develop TLC plates and jar for filter integrity test |
| †TLC Plate Reader | BioScan AR200,  Model B-AR-2000-1 | n/a | 1 | Radiochemical purity analysis, reader of TLC plates |
| †Endosafe Endotoxin Reader | Charles River Laboratories, Endosafe® nexgen-PTS^TM^, Model PTS150 | ASM-00019 | 1 | Bacterial Endotoxin Test (BET) |
| †Vortex Mixer | *Any vortex mixer will work* | n/a | 1 | Used to mix sample for endotoxin |
| Multi-Channel Analyzer | Canberra MCA,  Model 727 | n/a | 1 | Radionuclidic Purity analysis |
| **Disposable Items** | | | | |
| †**Used** 0.22 μm sterile product filter | Cathivex®-GV, 25mm diameter, 0.22 μm hydrophilic PVDF membrane, sterile | SLGV0250S | 1 | **Used filter from production** used to determine filter integrity |
| Silicone coated 18 G 1½” Needle | B. Braun Medical Sterican® Needles, sterile, 1.20x40 mm short bevel, Pink Hub | 4665120 | 1 | Needle to pull up QC aliquot from dose vial |
| 1 mL Organic Syringe | Norm-Ject®, 1 mL Luer Slip Tip Syringe, sterile, translucent plunger | NJ-9166017-02 | 1 | Syringe used to pull up QC sample |
| HPLC Vial | Chromacol,  300 μL fixed insert vial, 9 mm screw top | 03-FISV | 1 vial, ~ 0.1 mL sample | Vial used to hold QC sample |
| HPLC Vial Cap | SureStart^TM^, screw 9 mm yellow cap, white silicone, blue PTFE septa slit 1 mm | 6PSC9STS1Y | 1 cap | Vial cap for HPLC vial |
| TLC Plates | Agilent Technologies,  Glass microfiber chromatography paper impregnated with silica acid, 7 cm tall, spot sample 1 cm from bottom and develop to 1 cm from top | A120B12 | 2 plates, 1 μL sample/ plate | Used to analyze radiochemical purity |
| TLC Solution | 1:1 (1M) Ammonia Acetate (aqueous): methanol | n/a | ~ 6 mL | Solution used to develop TLC plates |
| pH indicator strips | Supelco, MQuant, non-bleeding, colorimetric pH indicator strips from 2.5 to 4.5 in 0.3 intervals | 109541 | 1 strip, 17.5 μL sample | pH indicator test |
| †Endosafe PTS Cartridge | Charles River Laboratories, The Endosafe®-PTS | PTS20F | 1, 1:300 dilution | Cartridge used to load samples |
| †8 mL Round-Bottom falcon tube | Falcon, 8 mL Polystyrene Round-Bottom Tube, 13x100 mm | 352027 | 1 | Used to hold sample in LAL water |
| †LAL reagent water | Charles River Laboratories,  Endosafe® LAL reagent water | W110 | 3.9 mL, 17.5 μL sample | Used to dilute sample to load into Endosafe cartridge |
| †Tryptic Soy Broth (TSB) | Tryptic Soy Broth  Acc EP+USP 3080r-100p | 1464320100 | 1 tube (9 mL), 250 μL sample | Used for sterility monitoring |
| †Fluid Thioglycollate Medium (FTM) | Thioglycollate Med.  Acc EP 2171r-100p | 1462200100 | 1 tube (9 mL), 250 μL sample | Used for sterility monitoring |

†Items marked with this symbol, are not required if following state board of pharmacy/ package insert rules and regulations and not cGMP under a PET manufacturing facility.

LAL – Limulus amoebocyte lysate, QC- quality control, rTLC – (radio) thin layer chromatography

**Preparation of TLC Eluent**

To make a 1 L batch of the TLC solution (1:1 (1 M) ammonium acetate solution (aqueous): methanol), 38.54 g of ammonium acetate is added into a clean 1 L bottle. 500 mL of Milli-Q water is added into the bottle and swirled until the ammonium acetate is dissolved. 500 mL of methanol is then added into the 1 L bottle and the bottle is inverted to fully homogenize the solution.


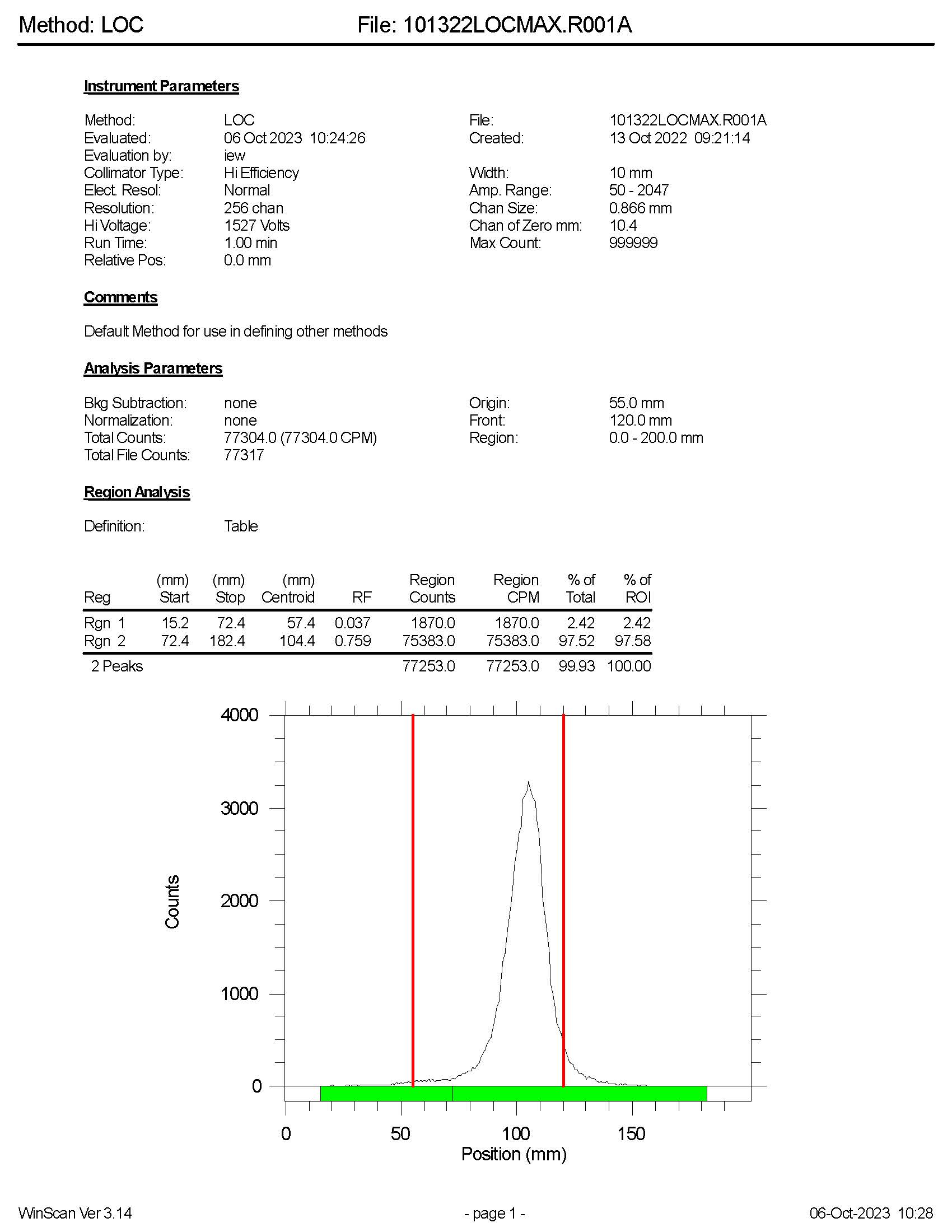
**Figure S1a**: rTLC of Fractionation Approach using Locametz, initial


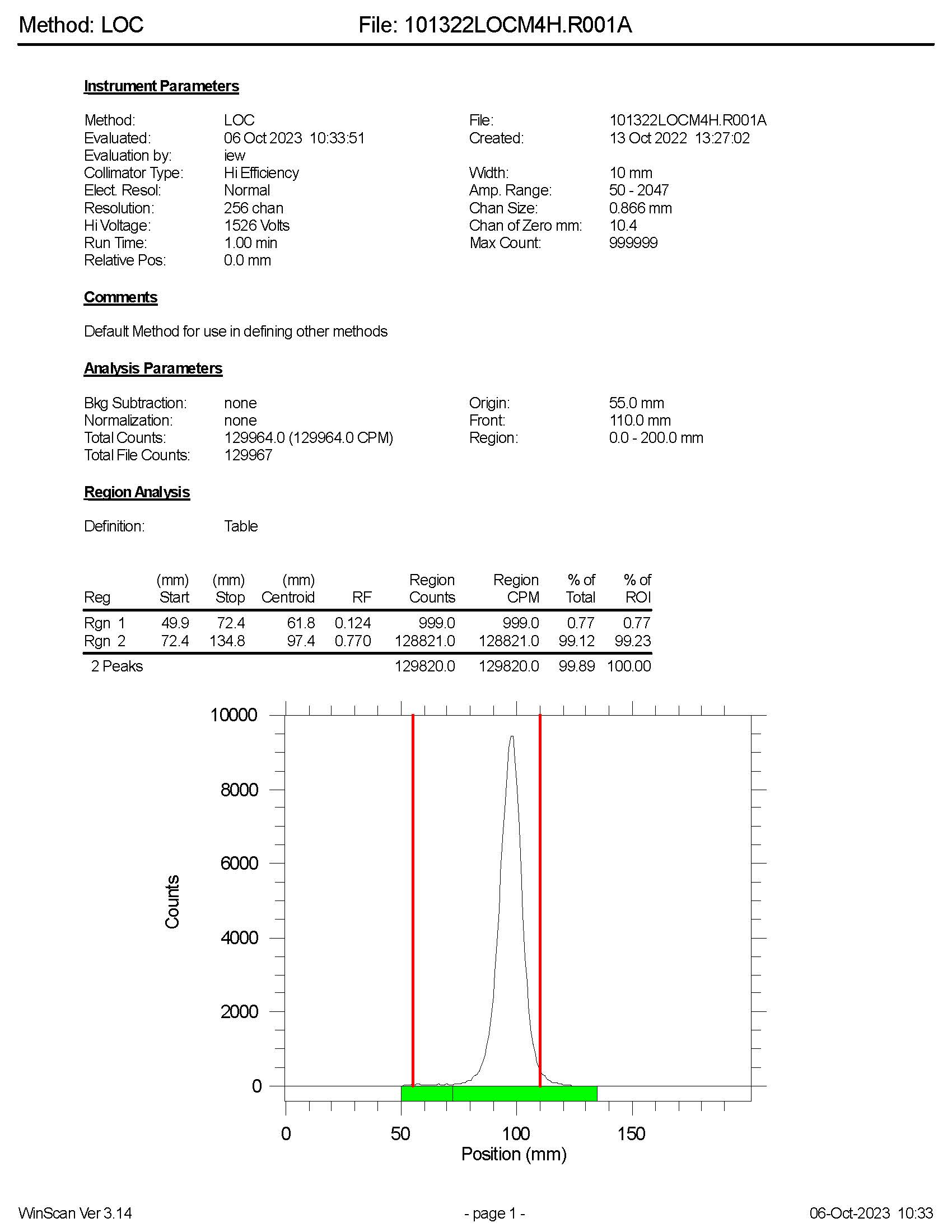
**Figure S1b**: rTLC of Fractionation Approach using Locametz, 4-hour stability


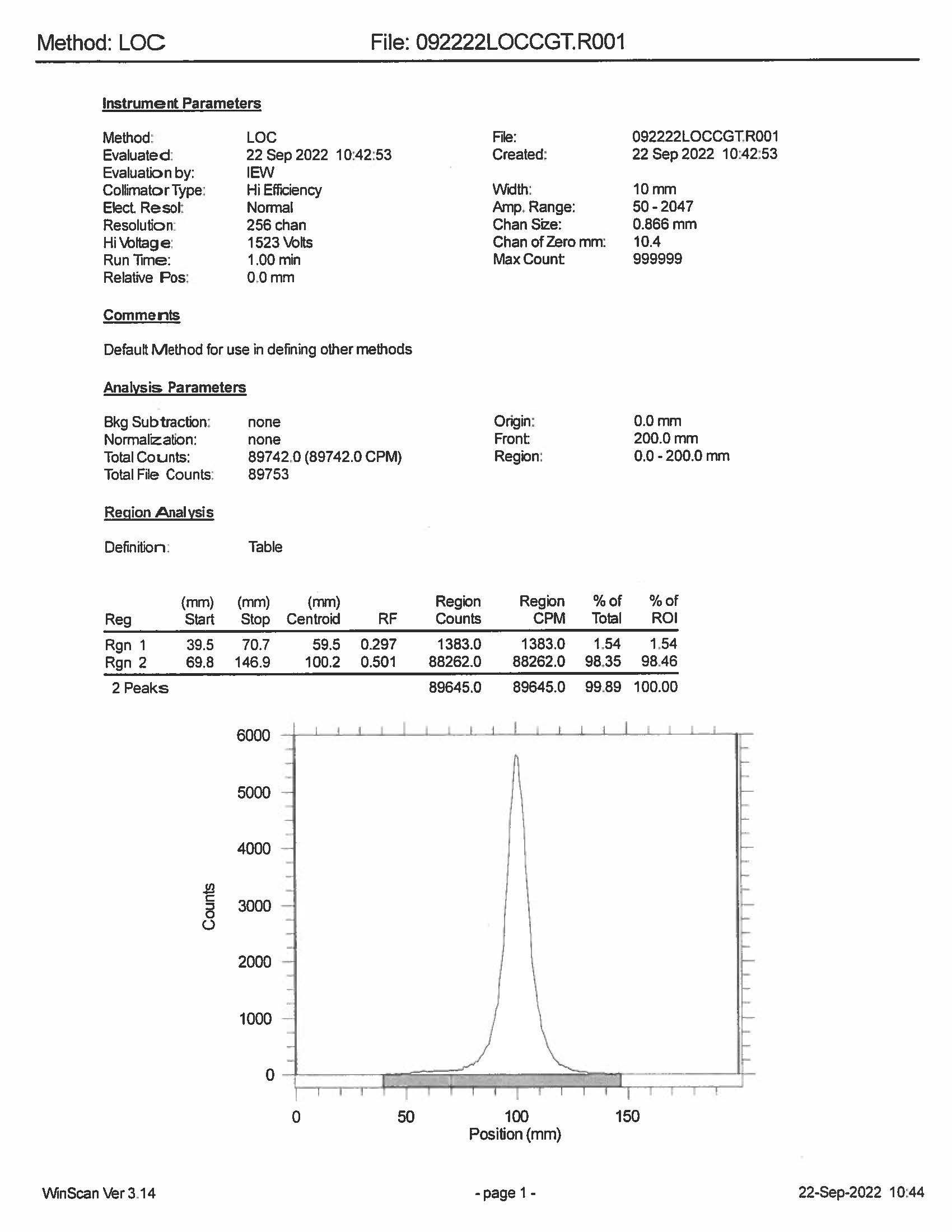
**Figure S2a**: rTLC of initial 3.7 GBq generator test using Locametz, initial


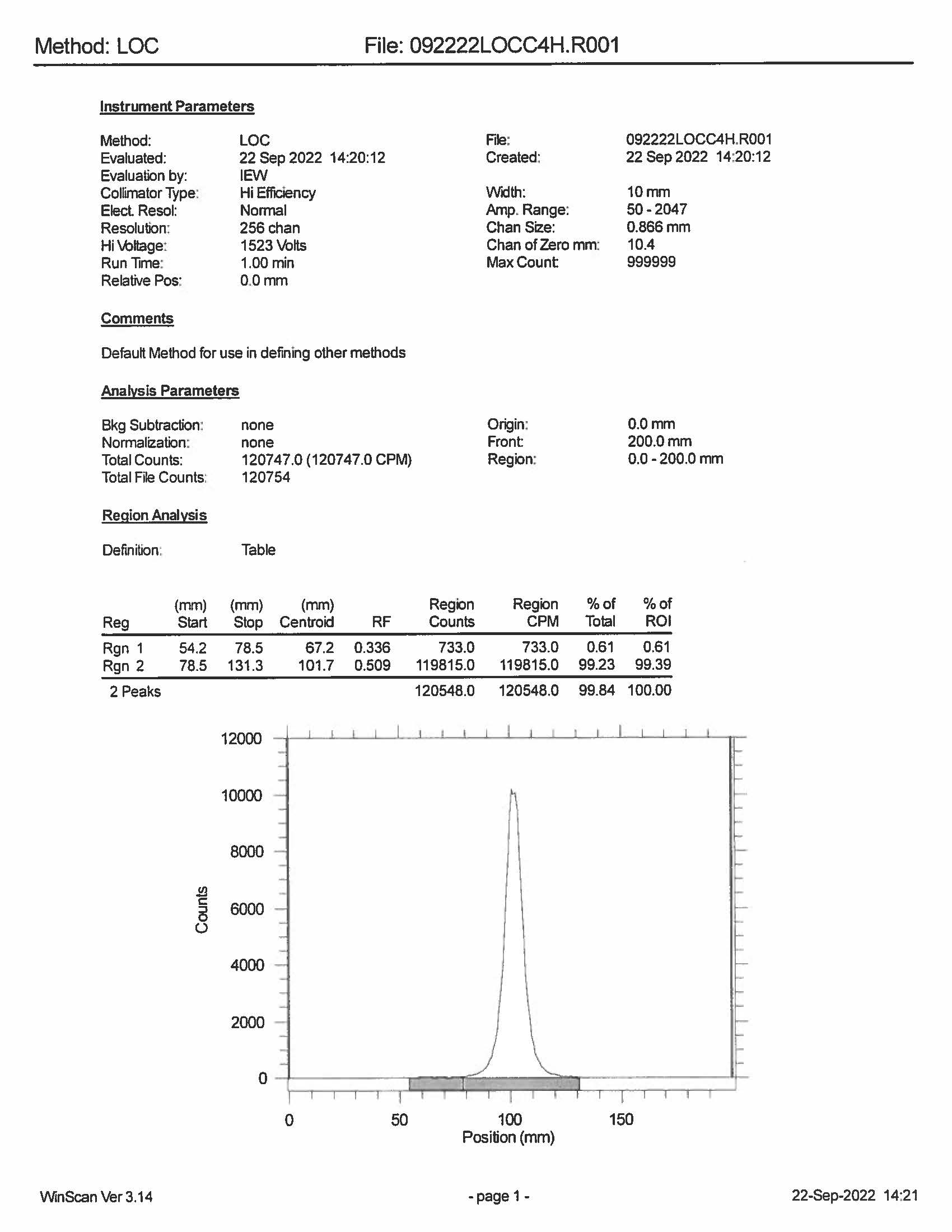
**Figure S2b**: rTLC of initial 3.7 GBq generator test using Locametz, 4-hour stability
